# Supplementary figures and images for: Large scale sequence-based screen for recessive variants allows for identification and monitoring of rare deleterious variants in pigs
Source: PLoS Genet. 2024 Jan 10;20(1):e1011034. doi: 10.1371/journal.pgen.1011034 (PMC10805306; doi:10.1371/journal.pgen.1011034)

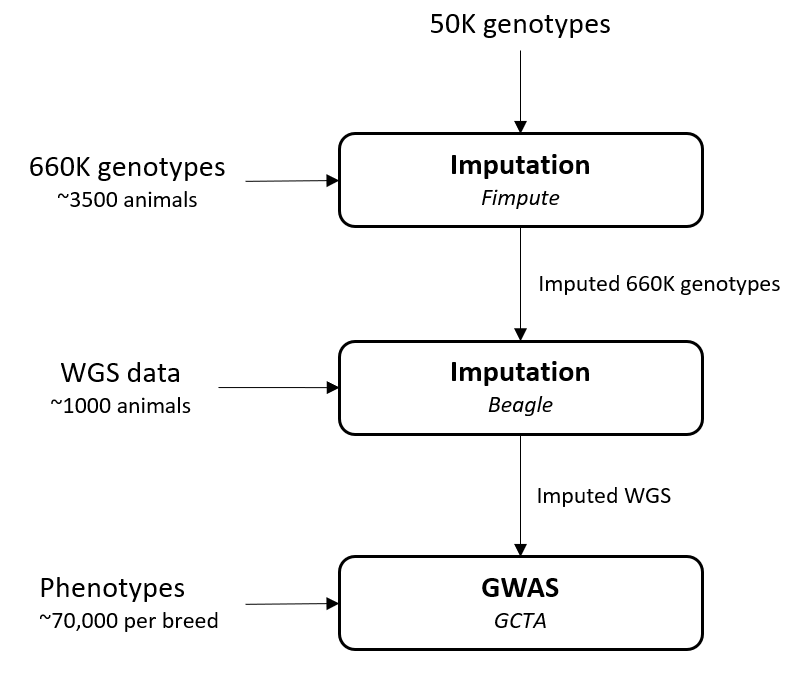

Supplement: S1 Fig — (TIF) [file pgen.1011034.s001.tif]

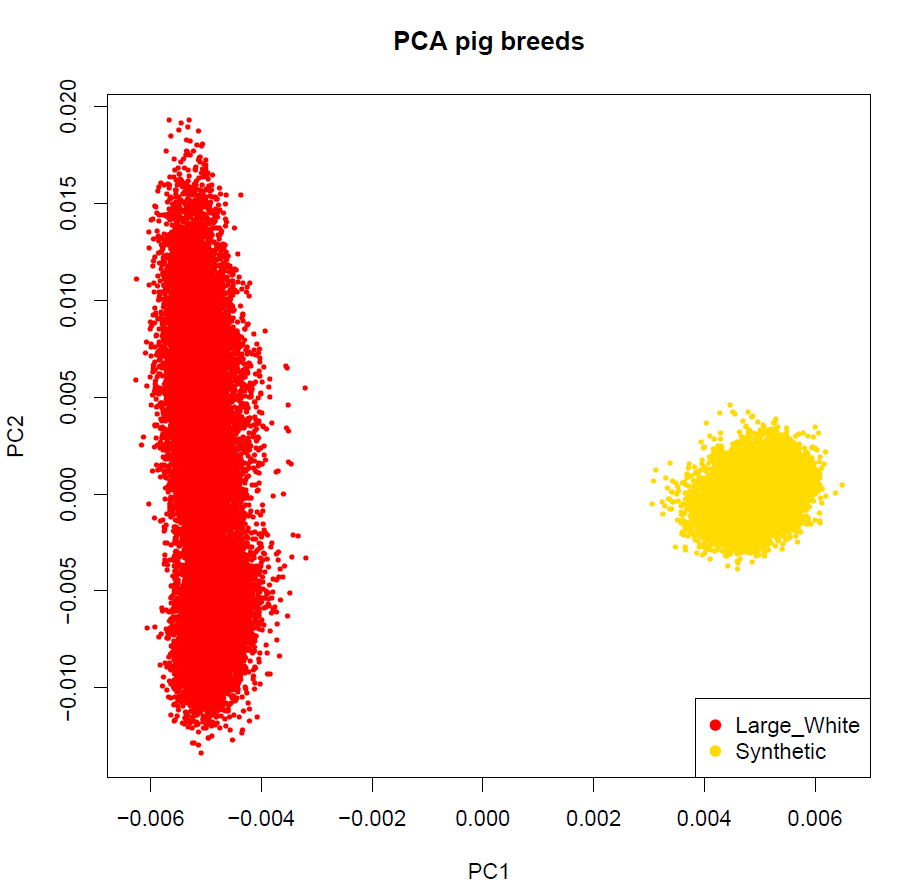

Supplement: S2 Fig — (TIF) [file pgen.1011034.s002.tif]
