# Supplementary material for: Large scale sequence-based screen for recessive variants allows for identification and monitoring of rare deleterious variants in pigs
Source: PLoS Genet. 2024 Jan 10;20(1):e1011034. doi: 10.1371/journal.pgen.1011034 (PMC10805306; doi:10.1371/journal.pgen.1011034)
Supplement: S1 Table — (PDF) [file pgen.1011034.s003.pdf]

S2 Table: Top SNPs for all QTLs resulting from the GWAS.
White = additive, blue = non-additive.

Chr	Pos	A1	A2	B	SE	P	R^2	AF	pCADD	Consequence	Gene
**Growth rate**											
**Large White**											
1	150364747	T	C	-10,62	1,50	1,57E-12	0,99	0,293	-	Intergenic variant	-
1	154978577	T	C	10,58	1,42	1,08E-13	0,95	0,354	-	Intergenic variant	-
1	159788889	C	T	-14,03	1,43	1,30E-22	0,99	0,101	7,986	Intron variant	*ENSSSCG00000034988*
1	160883673	T	C	8,27	0,98	2,86E-17	1	0,35	-	Intergenic variant	-
1	162020747	A	G	-11,64	1,23	1,71E-20	0,96	0,107	2,454	Intergenic variant	-
1	164838503	C	T	6,67	1,01	3,58E-11	0,98	0,444	0,330	Intergenic variant	-
2	60441882	A	C	12,24	1,79	8,35E-12	0,99	0,056	1,868	Downstream gene variant	*NR2F6*
2	79261674	A	G	-18,16	1,51	3,62E-33	0,99	0,104	1,009	Intron variant	*ADAMTS2*
2	81336954	T	C	-14,23	1,46	2,47E-22	0,99	0,103	22,615	Missense variant	*CDHR2*
5	66103958	A	G	7,70	0,89	4,63E-18	0,97	0,215	1,207	Intron variant	*CCND2*
6	146129928	T	A	-7,38	0,99	7,12E-14	0,95	0,142	4,272	Intergenic variant	*LEPR*
10	30278047	C	T	5,51	0,75	1,70E-13	0,98	0,396	7,647	Intron variant	*NTRK2*
10	30463237	AT	A	-6,57	0,92	1,26E-12	0,99	0,201	0	Intergenic variant	*NTRK2*
12	24571470	C	CT	-4,04	0,58	2,69E-12	0,66	0,258	-	Intron variant	*SKAP1*
13	50832581	A	G	-3,82	0,57	2,17E-11	1	0,326	-	Intron variant	*FRMD4B*
**Synthetic**											
1	159890563	T	C	-11,92	1,84	9,10E-11	1	0,223	-	Intron variant	*CDH20*
1	161332334	G	T	-18,62	2,39	7,56E-15	1	0,927	2,629	Intron variant	*CCBE1*
1	162878687	A	G	-11,31	1,82	5,72E-10	0,64	0,071	0,932	Intergenic variant	-
5	66103958	G	A	-7,28	1,12	1,02E-10	0,93	0,811	-	Intron variant	*CCND2*
6	55062615	T	C	-6,93	0,70	2,57E-23	0,97	0,642	-	Intron variant	*IZUMO2*
7	24913382	TC	T	-5,63	0,80	1,60E-12	0,95	0,276	0	Intron variant	*SLA-DRB1*
7	23835601	A	G	-5,63	0,80	2,42E-12	1	0,281	24,424	Missense variant	*MPIG6B*
13	68979390	C	T	-4,03	0,56	5,55E-13	0,95	0,529	10,189	Intron variant	*PLXND1*
15	121500039	T	C	-56,48	4,18	1,49E-41	0,97	0,044	13,565	Upstream gene variant	*SPEGNB*
**Backfat**											
**Large White**											
1	159794565	G	A	-0,23	0,03	7,56E-16	0,98	0,099	4,409	Downstream gene variant	*ENSSSCG00000034988*
1	160883673	T	C	0,16	0,02	1,60E-17	1	0,354	-	Intergenic variant	-
2	2354811	A	G	0,89	0,07	1,46E-38	1	0,004	4,182	Intron variant	*NADSYN1*
2	79236726	A	G	-0,25	0,03	1,25E-20	0,97	0,103	5,700	Intron variant	*ADAMTS2*
2	81336954	T	C	-0,20	0,03	3,70E-14	0,99	0,103	22,615	Missense variant	*CDHR2*
5	66103958	A	G	-0,24	0,02	3,87E-44	0,97	0,215	1,207	Intron variant	*CCND2*
6	147172050	T	C	-0,12	0,02	8,02E-15	1	0,355	-	Downstream gene variant	*AK4 (LEPR)*
6	170101951	A	G	0,12	0,02	1,29E-11	0,95	0,162	2,943	Intron variant	*SCMH1*
7	115433827	A	G	0,11	0,02	2,51E-12	0,97	0,246	0,044	Intron variant	*PPP4R4*
10	25732857	A	G	-0,13	0,02	2,41E-13	0,91	0,468	0,380	3_prime_UTR_variant	*ZNF367*
12	12278928	C	T	0,11	0,02	3,52E-11	0,98	0,185	-	Intergenic variant	-
16	35305197	A	T	0,28	0,03	3,59E-20	0,98	0,098	0	Intron variant	*ANKRD55*
18	10657589	G	T	-0,15	0,01	4,25E-39	1	0,255	-	Intergenic variant	*KIAA1549*
**Synthetic**											

1	114428892	A	G	0,25	0,02	1,67E-25	1	0,685	0,499	Intron variant	*CGNL1*
1	161332334	G	T	-0,33	0,04	1,79E-16	1	0,927	2,629	Intron variant	*CCBE1*
1	270630147	G	A	0,21	0,03	2,70E-12	0,99	0,024	4,293	Intergenic variant	-
5	66103958	G	A	0,18	0,02	5,84E-29	0,93	0,812	-	Intron variant	*CCND2*
10	25713012	A	G	0,17	0,02	9,60E-25	1	0,425	1,124	Intron variant	*ZNF367*
11	7867966	C	T	0,16	0,02	1,06E-20	1	0,581	-	Intron variant	*B3GLCT*
15	104968991	T	C	0,14	0,01	5,45E-37	1	0,557	-	Intron variant	*ALS2CR12*
15	121500039	T	C	1,27	0,05	6,91E-154	0,97	0,044	13,565	Upstream gene variant	*SPEGNB*
16	35245909	A	G	-0,20	0,01	1,87E-59	1	0,191	27,451	Stop gained	*ANKRD55*
18	10510398	T	G	-0,21	0,01	1,62E-135	1	0,315	0,477	Upstream gene variant	*IFT56*
