# Supplementary material for: Large scale sequence-based screen for recessive variants allows for identification and monitoring of rare deleterious variants in pigs
Source: PLoS Genet. 2024 Jan 10;20(1):e1011034. doi: 10.1371/journal.pgen.1011034 (PMC10805306; doi:10.1371/journal.pgen.1011034)
Supplement: S2 Table — (PDF) [file pgen.1011034.s004.pdf]

S3 Table: Validation of causal 5bp *OBSL1* deletion.

Labnr	Sample type	Sample name	Litter	conc. (ng/ul)	260/280	230/260	Allele 1	Allele 2	Conclusion
1	earpunch	Pig01	Litter1	599,3	1,8	1,5	259	264	heterozygous 5bp deletion
2	earpunch	Pig02	Litter1	623,1	1,8	1,6	259	264	heterozygous 5bp deletion
3	earpunch	Pig03	Litter1	584,1	1,8	1,5	259	264	heterozygous 5bp deletion
4	earpunch	Pig04	Litter1	599,1	1,9	1,7	264	264	
5	earpunch	Pig05	Litter1	558,2	1,9	1,6	264	264	
6	earpunch	Pig06	Litter1	551,2	1,8	1,6	259	264	heterozygous 5bp deletion
7	earpunch	Pig07	Litter1	645,9	1,9	1,7	259	264	heterozygous 5bp deletion
8	earpunch	Pig08	Litter1	578,3	1,8	1,7	264	264	
9	earpunch	Pig09	Litter1	572,3	1,9	1,6	264	264	
10	earpunch	Pig10	Litter2	625,8	1,8	1,7	264	264	
11	earpunch	Pig11	Litter2	757,0	1,8	1,6	259	264	heterozygous 5bp deletion
12	earpunch	Pig12	Litter2	659,3	1,8	1,5	264	264	
13	earpunch	Pig13	Litter2	600,8	1,8	1,6	264	264	
14	earpunch	Pig14	Litter2	590,7	1,9	1,7	259	264	heterozygous 5bp deletion
15	earpunch	Pig15	Litter2	646,3	1,8	1,6	264	264	
16	earpunch	Pig16	Litter2	533,8	1,9	1,6	264	264	
17	earpunch	Pig17	Litter2	521,9	1,8	1,5	259	264	heterozygous 5bp deletion
18	earpunch	Pig18	Litter2	635,4	1,8	1,6	259	264	heterozygous 5bp deletion
19	earpunch	Pig19	Litter3	550,5	1,9	1,6	264	264	
20	earpunch	Pig20	Litter3	580,0	1,9	1,7	259	264	heterozygous 5bp deletion
21	earpunch	Pig21	Litter3	667,9	1,8	1,7	264	264	
22	earpunch	Pig22	Litter3	688,5	1,9	1,6	264	264	
23	earpunch	Pig23	Litter3	639,1	1,8	1,5	264	264	
24	earpunch	Pig24	Litter3	562,1	1,9	1,6	264	264	
25	earpunch	Pig25	Litter3	607,9	1,8	1,6	259	264	heterozygous 5bp deletion
26	earpunch	Pig26	Litter3	604,8	1,8	1,6	264	264	
27	earpunch	Pig27	Litter3	535,2	1,8	1,5	259	264	heterozygous 5bp deletion
28	earpunch	Pig28	Litter3	692,1	1,8	1,6	259	264	heterozygous 5bp deletion
29	earpunch	Pig29	Litter3	651,3	1,8	1,5	264	264	
30	hair roots	Boar1	-	26,2	1,9	2,7	264	264	
31	hair roots	Sow1	-	11,0	1,8	2,0	259	264	heterozygous 5bp deletion
32	hair roots	Sow2	-	73,0	2,0	2,3	259	264	heterozygous 5bp deletion
