# Supplementary material for: Large scale sequence-based screen for recessive variants allows for identification and monitoring of rare deleterious variants in pigs
Source: PLoS Genet. 2024 Jan 10;20(1):e1011034. doi: 10.1371/journal.pgen.1011034 (PMC10805306; doi:10.1371/journal.pgen.1011034)
Supplement: Attachment — Submitted filename: Response_to_reviewers.docx [file pgen.1011034.s005.docx]

**Comment on data availability:**

In response to your request to provide details on the data availability we would like to point out that:

1) All imputed sequence data for the results discussed is provided in open science framework (200,000 animals in total!): <https://osf.io/fu6z5/> This repository is also public, and long-term funding is assured, thereby guaranteeing that the data availability meets present standards of being available for at least 5 years.

2) The reference population (1069 whole genome sequenced individuals) used for imputation is also added to the repository.

3) The complete raw GWAS output for all significant QTLs mentioned in the study, spanning 1MB up- and downstream from the top SNPs are provided, this included effect sizes (effect on phenotype) significance values etcetera.

The data combined will ensure that the research community can reproduce our results, according to current best scientific practice. We would like to point out that this is a truly unique dataset in volume, provided by Topigs Norsvin, a leading pig breeding company. The data pertains their two elite breeding lines, and essentially includes every selection candidate of the past years.

The unique data set accompanying the paper - which we strongly believe merits publication in PLoS Genetics even aside from the data availability - will hopefully spark further discussion in the animal breeding community regarding genotype data availability for scientific study. Data ownership concerns by breeding companies are currently precluding optimal academic use of the biggest and most expensive 'genetic experiment' on earth, i.e. the selection 'experiments' carried out by commercial breeding companies. Topigs-Norsvin are at the forefront of this discussion. We believe that Topigs-Norsvin are really providing a unique opportunity to demonstrate best scientific practices in academic-private partnership, which we hope will encourage other breeding companies to do the same in the future.

We are confident that our proposed data availability strategy meets PLoS high standards.

For the responded comments, line numbers refer tot the revised manuscript including mark-up.

Reviewer #1: I only have some very minor comments. This is an impressive study that addresses a difficult problem in livestock genomics and other species, capturing effects of low frequency variants.

We are very pleased with your positive feedback. We addressed your comments and believe that the manuscript has substantially improved based on your and the other reviewers feedback.

Page 8, line112; novel low “frequency” deleterious alleles Corrected
Page 12, line 168; We “found” a very significant Corrected
Page 14, lines 213-217; could you provide the rs# for these SNP if known? Now provided
Page 14, line 218; In heading, Low “frequency” additive QTL Corrected
Page 14, line 220; These SNPs have very low “frequencies” with MAFs Corrected
Page 17, line 272; low “frequency” deleterious variants Corrected
Page 17, line 282; lower “frequency” SNPs Corrected
Page 18, line 314; gene being fully “defective”. Corrected
Page 21, lines 376-380 or page 23, lines 429-431; could you describe the animal populations in more detail? We now provide more information on the lines (sow and boar) and the birth years of the animals. (Lines 446-448)

Page 21, line 390; could you describe the sequencing in more detail, i.e., coverage, read length etc Read length and average coverage and mapping quality have been added in the methods (Lines 401-402). Additionally, a full overview of coverage and mapping quality per sample is now available on OSF.

Page 23, line 427; do these gene expression datasets have accession numbers? Describe how SNP were associated with expression in more detail. The expression data was analysed according to the following conference paper: *M. Gòdia, M.F.L. Derks, B. Harlizius, O. Madsen, and M.A.M. Groenen. Proceedings of 12th World Congress on Genetics Applied to Livestock Production (WCGALP). 2022, 2313-2316*. In short, this is an eQTL study in pigs by integrating genomics, transcriptomics data from 100 crossbred animals. Four tissues (liver, spleen, lung, and muscle) were analyzed. RNA extraction utilized the Rneasy kit, followed by library preparation with NEBNext® Ultra™ Directional RNA Library Prep Kit, and sequencing on an Illumina 6000 platform. Bioinformatic analysis involved trimming, alignment to the Sus scrofa genome, gene counts assessment, and TMM normalization. Genomic data were obtained using the Axiom™ Porcine Genotyping Array. The study performed expression GWAS (eGWAS) and identified significant associations. The data is not yet publicly available but will soon become available along with the publication of this study.

Page 23, line 428; dye-labeled with “either”? ‘Either’ removed

Although not essential, recent reports have also identified a few of the candidate genes you report for growth and fatness QTL and could be included and discussed.
Shi L, Wang L, Fang L, Li M, Tian J, Wang L, Zhao F. Integrating genome-wide association studies and population genomics analysis reveals the genetic architecture of growth and backfat traits in pigs. Front Genet. 2022 Nov 25;13:1078696. doi: 10.3389/fgene.2022.1078696. PMID: 36506319; PMCID: PMC9732542.
Heidaritabar M, Bink MCAM, Dervishi E, Charagu P, Huisman A, Plastow GS. Genome-wide association studies for additive and dominance effects for body composition traits in commercial crossbred Piétrain pigs. J Anim Breed Genet. 2023 Jul;140(4):413-430. doi: 10.1111/jbg.12768. Epub 2023 Mar 7. PMID: 36883263. Thank you for sharing, I’ve included a section in the discussion where I discuss these findings. (Lines 366-370)

Reviewer #2: The authors used a large-scale pig data set with in total 200k genotyped and phenotyped sows to detect recessive deleterious variants. The genomes of the individuals are imputed up to whole genome sequence-level genotypes. The applied GWAS approach is straightforward and comparable simple, but it was successfully applied and the results are very interesting and discussed in detail. The paper is very well written. In summary, I would classify it as a higher scientific content study and as such it fits well to the Journal.

We are very pleased with your positive feedback. We addressed your comments and believe that the manuscript has substantially improved based on your and the other reviewers feedback.

I have a couple of minor remarks.
1. The data sets were analysed separately to contrast the results between the Large White and Synthetic data set. The question is why, in addition, a joint analysis was not conducted to utilize the full power to detect the recessive alleles. The joint analysis could be applied using summary data already generated within the data sets by applying a meta-analysis. We think the datasets are large enough to look at the breeds separately, but for further analysis performing a meta-GWAS could be interesting, to even have increased power. However, we don’t expect to obtain many novel results in this way, which is why we have not done this initially. In addition, we were struggling computationally to analyze and perform a sequence based GWAS on >100,000 individuals at once. Although we can use the statistics that come out of the individual GWAS for a meta-GWAS we did not apply this. Moreover, most of the significant low frequency non-additive variants are breed-specific and will likely not benefit from doing a meta-GWAS.

2. Line 422: ‘the non-additive model GWAS, all heterozygote genotypes were set to 0/0 to test the phenotypes of wildtype and heterozygous animals against homozygous animals‘. It is not clear how the wild type genotype was identified. In addition, why not applying an additive and a dominance effect simultaneously to the model? This would result in one extra parameter estimate and the power of the experiment should be high enough to detect the main recessive effects also under this parametrization. The major alleles were set as reference allele. Animals homozygous for this allele are considered wildtype, I have made this more clear in the mentioned line (438). We ran the models separately as the software we use can only run one model at a time, which was already pushing the computational limits.

3. How were the indels modeled under the applied GWAS parametrization? We treated indels the same as SNPs, we don’t think that modelling the indels separately will gain a significant amount of power.

4. No population stratification effects on the GWAS results are shown. I suggest to add a lambda value and / or a qq plot to show some putative inflation of significant hits. We now include a PCA analysis to show population stratification across the two breeds (Supporting Figure 4). Note that we did correct/account for population stratification in our GWAS analysis through pre-correcting the phenotypes for non-genetic effects as mentioned in line 445.

5. Imputation accuracy is an important parameter for these kind of analysis with a focus of rare variants. How was this evaluated in detail? This becomes not clear at present. This was evaluated based on the R2 value that Beagle outputs for each imputed SNP, which is the squared correlation between the true number of non-major alleles on a haplotype an the posterior imputed allele probability. These values are evaluated and presented under ‘Imputation to Sequence’ on page 5. I have added these extra details in the methods (Lines 423-425).

6. I’m a bit surprised by he very clear signals spanning a low genomic region. This must have been a result from a very high mapping resolution, i.e. s strong LD decay. This is partly shown in Table 2, but a genomewide illustration 7 analysis of the LD structure would be helpful to understand the high mapping resolution. Yes we agree we have high mapping resolution which is partly also the consequence of the size of the dataset >100,000 individuals. However, there seems to be indeed an interesting LD structure in this region. We expect this is due to the SNPs tested being located on different haplotypes.

7. A bit more explanation is needed to understand Figure 5A. A more detailed explanation is now provided.

Reviewer #3: Thanks for the interesting manuscript entitled 'Large scale sequence-based screen for recessive variants allows for identification and monitoring of rare deleterious variants in pigs'. It was very well written. In following you can see my comments and questions:

We are very pleased with your positive feedback. We addressed your comments and believe that the manuscript has substantially improved based on your and the other reviewers feedback.

1) Please explain, why you have chosen only these two traits i.e., growth rate and backfat thickness?
Why these two traits are important to find novel QTLSs? What about the other traits such as feed consumption, loin muscle thickness, intramuscular fat or the other production traits? As this was the first time we have tried both GWAS on a sequence level scale and running a non-additive model, we first wanted to perform this analysis on a small number of non-complex traits. Additionally, we wanted to use traits where we had a large number of phenotype data available. Because the power of detecting deleterious recessive variants lies with a very large sample size. In addition, for other production traits like intramuscular fat and also fertility traits the datasets (phenotypes) are considerably smaller, but are growing rapidly.

2) Figure 2: Am I correct? The significant SNPs for BF on chromosome 1: 224 Mb were found for synthetic breed. In figure 4, I see two blocks for this region for Large White pigs. Similar for chromosome 2: 2 MB two blocks for BF for synthetic breed. Is it not supposed to be for Large White pigs? I assume the legend are reverse. Up is for synthetic breed and down is for Large White breed. The legend was indeed reversed, thank you for pointing this out. It is now corrected.

3) Lines 315-320: Did you have some homozygous animals for this QTL on Chr15? That could the expression of OBSL1 gene is not like human or even sheep. Based on the study in sheep, lambs with hypoplasia syndrome were stillborn. There were homozygous animals in our dataset, but none of these are alive anymore. We checked for matings leading to potential homozygous offspring but did not find any, so we were not able to observe any homozygous animals. This is discussed in lines 327-330.

4) Line 321: Please mention the position of this SNP in the Synthtic breed (Chromosome 7: 23.8 Mb). Chromosome number is now mentioned (Line 331)

5) Line 331-333: Monitoring and avoidance of crossbred mating can be suggested only for this SNP? This is the only significant SNP we found that could segregate through crossbred mating, but of course monitoring and avoidance can be suggested for all variants with high effects. This is now made more clear in the text. (Lines 343-344)
